# In-Solution Conformational Analysis of the XCYCH_3_ Moiety for Small Esters and Ethers with all Combinations of X, Y = O, S

**DOI:** 10.3390/molecules18078063

**Published:** 2013-07-08

**Authors:** Peter I. Nagy

**Affiliations:** Center for Drug Design and Development, the University of Toledo, Toledo, OH 43606, USA; E-Mail: pnagy@utnet.utoledo.edu

**Keywords:** B97D/aug-cc-pvtz, IEF-PCM, *cis*-trans conformation equilibrium, atomic charges

## Abstract

Favorable steric and electrostatic fit of a ligand to a receptor is of central interest in theoretical drug design. This paper considers the effects of non-protic solvents, in comparison with the gas phase, on the preferred conformation of the XCYCH_3_ moiety of simple aliphatic esters and heterocyclic methyl ethers with all combinations of the X and Y atoms as oxygen and sulfur. An IEF-PCM/B97D/aug-cc-pv(t+d)z continuum dielectric solvent study in chloroform and acetonitrile explores the through-space polarization effect of the environment on the conformational preference, not affected by possible solute-solvent hydrogen bond formation. The inherently favored structure for the present molecules is important, since the hypothetical oxygen and sulfur lone-pairs point approximately in opposite directions in the cis conformation of esters, whereas the trans and gauche conformations for the methyl group in ethers define nearly parallel or perpendicular directionality for the lone pairs of the ring heteroatoms and the O or S atoms connecting to the ring. These different preferences for the studied two families of compounds allow for designing formation of hydrogen bonds with a protein in fairly different regions of the latter still within the ligand-binding cavity. For a fine-tuning of these hydrogen bonds, a replacement of an oxygen atom of the ligand by a sulfur atom could be a straightforward possibility.

## 1. Introduction

One of the major problems throughout the theoretical design of a drug candidate is the choice for a ligand structure that would optimally fit to the targeted receptor. Optimal fit is meant here as the best compromise for the steric and electrostatic complementarity in the ligand-biomacromolecule complex, as well as the matching of their hydrophobic sites [1]. By means of the structure based design (SBD), the procedure becomes feasible if the tertiary structure of an enzyme or a protein receptor is known, which is more and more available recently on the basis of their determined crystal structures.

Esters and ethers are frequently appearing substructures in drug molecules. In the common oxo-esters there are two oxygen atoms acting as possible hydrogen-bonding acceptor sites. Heterocyclic ethers with an O-C-O substructure (see 2-OCH_3_ furan in the present study) could be an alternative for the O=C-O moiety in a drug candidate. Previous investigations predicted that both types of molecules are good hydrogen bond acceptors [2,3,4]. For ester/ether ligands, a possible way for fine-tuning the hydrogen bond is the replacement of an oxygen atom by a sulfur atom. There are four possible combinations when O and S atoms are considered in an XCYCH_3_ moiety, namely XY = OO, OS, SO, and SS. The most important issue in this respect is that the favored conformation in the XCYCH_3_ moiety could be more or less different. Diversities in the molecular geometries, lone-pair orientations and atomic charges could help optimizing the ligand-protein interactions.

Correct determination of the relative conformational energies for drug candidates is the basic need in SBD. The primary question is: what are the reasonable theoretical method and level? Semi-empirical quantum chemical methods could provide useful information for some structures, whereas they fail properly working for other systems (see below). Thus the present theoretical study is a high-level conformational investigation of a simple aliphatic ester, methyl acetate, its thio- and dithio- analogues, as well as for the 2-OCH_3_ and 2SCH_3_ furan and thiophene ethers. Potential energy curves for the rotation about the C-Y bond in the gas phase were compared with those in chloroform and acetonitrile in order to characterize the effects of the solvent on the in-solution optimized molecular geometry and relative conformer energy. These solvents were chosen to avoid the possible effect of the solute-solvent hydrogen bond formation, thus the results reflect the “inherent” conformational preference of the solute in two, non-protic solvents with considerably different dielectric constants.

If one intends to compare conformational energies for a set of molecules, the same theoretical level and basis set are to be used. The goal of the present study is to point out the solvent effect on the relative conformer energies. Thus even if a number of gas-phase studies are available for some of the present eight molecules, they could be considered only for a partial comparison. Such comparison has become possible for CH_3_COOCH_3_ and CH_3_COSCH_3_, based on some former publications [2,5,6,7,8]. These papers also reported different solvent-effect calculations using different theoretical methods and basis sets. Thus they cannot serve as a basis for a consistent comparison regarding the present target molecules, mainly not since no solvent-effect calculations have been found in the literature for the conformational preferences of the 2-OCH_3_ and 2-SCH_3_ heterocyclic ethers. For furan and thiophene derivatives in solution, only some review works have been identified [9,10]. However, from the point of view of an SBD, relative free energies are meaningful only as existing in some environment, namely in a protein cavity that could be characterized by low, although inhomogeneous dielectric constant.

## 2. Methods and Calculations

**Figure 1:**
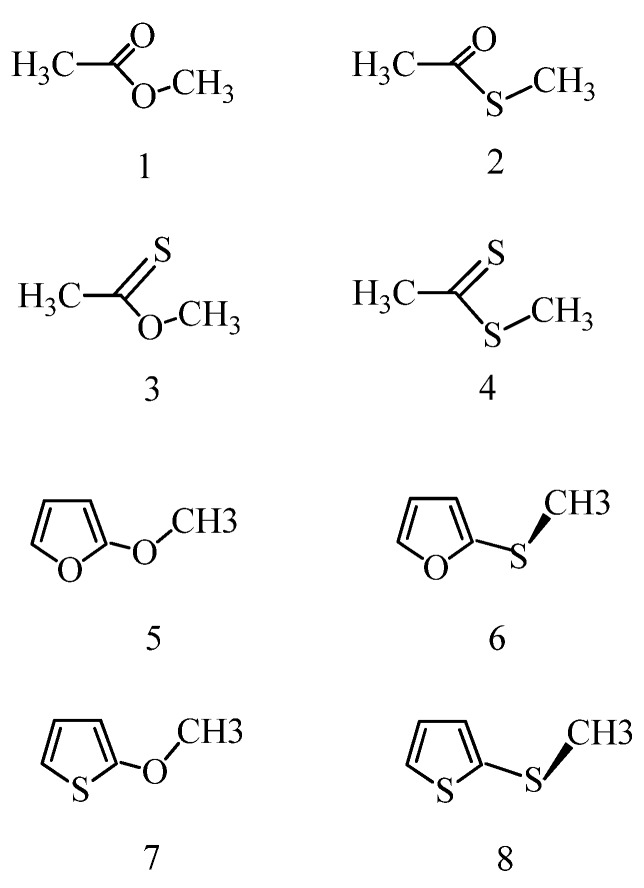
Compound structures: methyl acetate (**1**); S-methyl thioacetate (**2**); O-methyl thioacetate (O-methyl ethanethioate, **3**); dithioacetic acid methyl ester (**4**); 2-methoxyfuran (**5**); 2-methylthiofuran (**6**); 2-methoxythiophene (**7**); 2-methylthiothiophene (**8**). The indicated conformation of the esters **1**–**4** is the most stable *cis* form. For 2-methoxyfuran (**5**) and 2-methoxythiophene (**7**) the most stable form is *trans*, and for 2-methylthiofuran (**6**) and 2-methylthiothiophene (**8**) the most stable form is *gauche*.

Investigated species, structures **1**–**8** in their most stable conformations are shown in Figure 1. The calculations were performed by means of the Gaussian 09 package [11] running on the platform of the Ohio Supercomputer Center. Symmetry-unrestricted geometry optimizations in the gas phase were performed by means of the B97D density functional method [12] using the aug-cc-pvtz [13,14,15] basis set and aug-cc-pv(t+d)z basis set [16] for S containing molecules. Reoptimizations in chloroform and acetonitrile, with dielectric constants accepted as 4.71 and 35.7 at T = 298 K°, respectively, were performed by using the integral equation formalism of the polarizable continuum method, IEF-PCM [17,18] at the above-indicated theoretical level. Cavity radii for the C, O, S atoms were set to scaled Bondi values [19,20], and the united CH_3_ and CH group approach was applied in other cases [20]. This latter approximation has minimal effect on the optimized geometry [21]. Local energy minima were identified by all positive vibrational frequencies. The relative internal Gibbs free energy, ΔG_int_ is the sum of the relative internal energy, ΔE_int_ and the relative thermal correction, ΔG_th_, where ΔG_th_ was calculated in the rigid-rotor, harmonic-oscillator approach at T = 298 K° and p = 1 atm [22]. The relative solvent-effect related free energy contribution, ΔG(solv) was calculated as the sum of ΔG_elst_ for the relative solute-solvent electrostatic interaction free energy and of ΔG_drc_, standing for the relative solute-solvent dispersion + repulsion free energies, plus for the difference in the cavitation free energy upon the conformer transformation. ΔG_drc_ generally amounts only to about 0–0.2 kcal/mol, in comparison with ΔG_elst_ up to several kcal/mol in the present calculations. The total relative free energy is ΔG_tot_ = ΔE_int_ + ΔG_th_ + (ΔG_elst_ + ΔG_drc_) = ΔE_int_ + ΔG_th_ + ΔG(solv). Applied formulae were described earlier [23,24].

Optimized geometric parameters show a remarkable basis set dependence in general. It was revealed from recent calculations [23,24,25] that the calculated relative free energy in conformational/tautomeric equilibria is also subject to the basis set effect, even if the relative internal energies are calculated at the aug-cc-pvdz level. B97D/aug-cc-pvtz relative internal energies are, however, close to the complete basis set limit [23], thus ΔE and ΔG values were calculated at this level in the present study as nearly converged values. Nonetheless, the aug-cc-pvtz basis set still may not be large enough for S containing molecules. Calculated bond lengths to the sulfur atom in some heterocyles could be considerably ameliorated by considering “d” orbitals on S [26,27]. Accordingly, the aug-cc-pv(t+d)z basis set was eventually used in the present optimizations for the thio- dithio- ester analogues, the furan thiomethyl ether, and the thiophene derivatives. For comparison, some gas-phase calculations with geometry optimization were performed at the MP2 level [28] with the same basis set as used with B97D, and semi-empirical calculations at the PM3, PM6, and PDDG/PM3 levels [29,30,31] were carried out for some gas-phase CH_3_COSCH_3_ conformers.

Due to the large number of the needed structure optimizations and especially the lengthy determination of the TS (transition state) structures, torsional potential curves were approached upon considering only a limited number of torsion angles for the XCYC moiety. The energy for the top of barrier was approximated generally by the value calculated at XCYC = 90°. More precise torsion potential curves for CH_3_COOCH_3_ (structure **1**) and CH_3_CSSCH_3_ (structure **4**) (see below) support this approach.

## 3. Results and Discussion

When conformational equilibria are studied, *relative* energies/free energies are *always* to be considered. These values could be relevant even if the underlying optimized geometries and individual energy terms are only moderately correct. Thus equilibrium calculations could lead to valuable conclusions despite some possible systematic error in the applied theoretical method and/or due to the incompleteness of the basis set. If reasonable trends are noted for the individual species by applying gradually increasing theoretical level through the investigations, and the calculated relative values show convergence, the obtained results may be acceptable. Still, however, it is not easy to verify that the predicted relative free energies and the concomitantly derived equilibrium composition are correct.

In cases, experimental information is available regarding the structure and the equilibrium composition. Experimental data are mostly available for gas-phase systems, and generally only for the structure of the predominant conformer. Even less is known for in-solution equilibria and related conformer structures. Theoretical calculations become of paramount interest in these cases. Since no experimental data have been found for the in-solution structure and equilibrium of the eight target molecules in the present study, the trends for their conformational preferences may provide useful information regarding their behavior through in-solution reactions and interactions with protein receptors in a binding cavity.

### 3.1. Geometries

Optimized molecular geometric parameters are compared with available experimental values for furan and thiophene (Table 1) and for methyl acetate (structure 1) and thioacetic acid S-methyl ester (structure 2) (Table 2). The calculated bond distances were estimated in remarkably better accord with the experimental values when the aug-cc-pvTz basis set rather than the aug-cc-pvDz set was used (Table 1). However, the O-C distance in furan is still overestimated by 0.008 Å even when the larger basis set is used [32]. (From experimental data, the symmetry of the furan and thiophene molecules is C_2v_, thus symmetry-restricted geometry optimizations were performed in these cases.) For thiophene, the calculated S-C distance improves by more than 0.02 Å with increasing basis sets. The bond angles are in good agreement with the experimental values at any calculation level [33]. For the present purposes, however, the main goal is not achieving very high precision in individual geometries. As emphasized in the Introduction, this study aims to point out the trends of the changes in molecular conformations and relative free energies, affected by the solvent. Table 1 indicates negligible solvent effect for ring structures.

**Table 1:** C_2v_ symmetry restricted optimized geometries at the B97D/aug-cc-pvXz level for furan and thiophene ^a^.

	Gas			CHCl_3_	CH_3_CN	Exp ^b^
	X = D	X = T		X = T	X = T	
**Furan**						
OC	1.375	1.370		1.373	1.374	1.362
C_2_C_3_	1.373	1.363		1.363	1.364	1.361
C_3_C_4_	1.442	1.435		1.436	1.437	1.431
CH_α_	1.088	1.080		1.082	1.082	1.075
CH_β_	1.090	1.082		1.083	1.084	1.077
COC	106.8	106.7		106.7	106.7	106.6
OCC	110.4	110.4		110.3	110.3	110.7
CCC	106.2	106.3		106.3	106.4	106.1
OCH_α_	115.8	115.7		115.8	115.8	115.9
OCH_β_	126.3	126.3		126.3	126.3	128.0
	**X = D**	**X = T**	**X = T + d**	**X = T + d**	**X = T + d**	
**Thiophene**						
SC	1.745	1.730	1.723	1.724	1.725	1.714
C_2_C_3_	1.381	1.373	1.374	1.374	1.375	1.370
C_3_C_4_	1.434	1.426	1.426	1.427	1.427	1.423
CH_α_	1.091	1.082	1.082	1.084	1.085	1.078
CH_β_	1.094	1.085	1.085	1.086	1.087	1.081
CSC	91.6	91.9	92.1	92.2	92.2	92.2
SCC	111.4	111.4	111.4	111.3	111.2	111.5
CCC	112.8	112.7	112.6	112.6	112.7	112.5
SCH_α_	119.8	120.1	120.2	120.2	120.2	119.9
SCH_β_	123.2	123.2	123.3	123.3	123.2	124.3

^a^ Distances in Å, angles in degrees. Co-planar geometry, C_2v_ symmetry. ^b^ Gas-phase microwave data, refs. [32] and [33] for furan and thiophene, respectively.

Table 2, Table S1 summarize the calculated characteristic geometric parameters for simple esters. All results were obtained from symmetry-unrestricted optimizations starting from some structure of C_1_ symmetry. The symmetry of the converged *cis* and *trans* structures corresponds only *nearly* to C_s_, the torsional angles for the heavy atoms deviate from 0 or 180° by some hundredths of a degree (achievement of a perfect symmetry plane is almost impossible when the standard convergence thresholds are used in a symmetry-unrestricted energy minimization in Gaussian. The symmetry-unrestricted optimization assures, however, that the structure does not remain stuck in some transition state if the starting geometry is wrongly chosen; a possible source for defining a transiton state when the methyl group is set in an improper rotational position). It reveals from cases when comparison with experimental values is possible (Table 2) that the gas-phase B97D single bonds are overestimated by up to 0.02 Å for methyl acetate [34] and thioacetic acid S-methyl ester [35]. The largest overestimation was noted for the C-C bond, which probably has, however, minor effect on the conformational equilibria. For the thioacetic acid S-methyl ester, the C-S and S-C distances are too long by up to 0.016 Å. The calculated bond angles for the *cis* conformers agree within 1° with the corresponding experimental values.

**Table 2:** Geometric parameters optimized at the B97D/aug-cc-pvTz and B97D/aug-cc-pv(T+d)z levels for methyl acetate and thioacetic acid S-methyl ester, respectively ^a^.

	Gas	CHCl_3_	CH_3_CN	Exp.
CH_3_COOCH_3_, *cis* ^b^				
C-C	1.514(1.500)	1.511	1.510	1.496
C=O	1.212(1.212)	1.217	1.219	1.206
C-O	1.363(1.351)	1.357	1.354	1.357
TS	1.393	1.389	1.387	
O-CH_3_	1.448(1.437)	1.453	1.454	1.438
C-C=O	125.8(125.9)	125.6	125.6	
O=C-O	123.8(123.3)	123.6	123.6	123.0
C-O-CH_3_	115.4(114.1)	116.1	116.3	116.4
TS	114.6	114.5	114.5	
HCC=O	1.1(0.2)	0.6	0.5	
CH_3_COOCH_3_, *trans*				
C-C	1.519	1.514	1.511	
C=O	1.207	1.215	1.218	
C-O	1.370	1.361	1.358	
O-CH_3_	1.440	1.449	1.453	
C-C=O	124.0	124.1	124.2	
O=C-O	118.5	118.0	117.8	
C-O-CH_3_	120.4	120.3	120.3	
HCC=O	0.1	0.1	0.1	
CH_3_COSCH_3_, *cis* ^c^				
C-C	1.520(1.507)	1.517	1.516	1.499
C=O	1.213(1.216)	1.218	1.220	1.214
C-S	1.797(1.769)	1.791	1.788	1.781
rot 90°	1.885	1.883	1.883	
S-CH_3_	1.820(1.798)	1.820	1.820	1.805
C-C=O	123.4(123.6)	123.3	123.2	123.4
O=C-S	123.1(122.7)	123.1	123.1	122.8
C-S-CH_3_	99.9(98.5)	100.7	101.1	99.2
rot 90°	95.9	96.3	96.4	
HCC=O	180.0(178.8)	178.7	178.5	143.1
CH_3_COSCH_3_, *trans*				
C-C	1.518	1.514	1.511	
C=O	1.212	1.218	1.221	
C-S	1.805	1.796	1.793	
S-CH_3_	1.829	1.828	1.828	
C-C=O	123.9	124.0	124.0	
O=C-S	118.1	117.9	117.8	
C-S-CH_3_	105.1	105.2	105.3	
HCC=O	0.0	0.6	0.6	

^a^ Distances in Å, angles in degrees. Cis and trans structures correspond to O=C-X-C (X=O, S) torsional angles of very close to 0° and 180°, respectively, heavy atoms are *nearly* coplanar. Values in parentheses from MP2 optimization with the same basis set. B97D parameters with characteristic changes in the transition state geometry or for the X=C-Y-C = 90° structures (rot 90°) are indicated. ^b^ Experimental data from ref. [32]. Corrected geometric parameters based on gas electron diffraction experiment. ^c^ Experimental data from gas electron diffraction, ref. [33].

The most conspicuous changes of the geometric parameters upon the *cis* to *trans* transformation were noted for some bond angles. For the two esters in Table 2, the O=C-X angles decrease and the C-X-CH_3_ (X=O, S) bond angles increase by about 5° in the gas phase. For the C=S containing esters (Table S1) the S=C-X angles decrease by 7–8°, and the S-CH_3_ distance increases in the *trans* conformer of the dithioacetic acid ester by more than 0.02 Å.

The H-C-C=O torsion angles are about 0 and 180° for the *cis* CH_3_COOCH_3_ and CH_3_COSCH_3_ conformers, respectively, both at the B97D and MP2 levels. The experimental value for CH_3_COOCH_3_ was not provided by Pyckhout *et al*. [34]. The torsional angle is 143.1° (without refinement) for CH_3_COSCH_3_ from gas electron-diffraction measurements by Della Védova *et al*. [35]. This torsion angle was calculated at 180° and 178.8° using the B97D and MP2 method, respectively, and applying the aug-cc-pv(t+d)z basis set. It is remarkable that the H-C-C=O torsion angle changes to 0° for the *trans* conformer.

The solvent effects are small and consistent for any geometric parameters. By consistency the finding is meant that the geometric parameters monotonically decrease or increase with increasing polarity of the environment in the gas-phase, chloroform and acetonitrile series. Remarkable changes were found for some atom distances, which were larger by up to 0.017 Å in acetonitrile compared to those in the gas phase.

### 3.2. Energy Results for Esters

Relative energy/free energy components of ΔG_tot_ for the studied esters are compared in Table 3. Due to the practically achieved symmetry plane for the molecules in their *cis* and *trans* conformations, and the energy equivalence of the clockwise and anticlockwise rotation about the C-Y bond, these structures must correspond to either a local energy minimum or a transition state. Non-planar structures exist in two mirror image forms, and for their ΔG_tot_ value the entropy of mixing term, -RT ln 2 = −0.41 kcal/mol is to be considered. Table 3, Table 4 include the ΔG_th_ values for the local-energy- minimum and TS structures in both solvents, and accordingly the correct ΔG_tot_ = ΔE_int_ + ΔG_th_ + ΔG(solv) was calculated. For torsional rotamers corresponding neither to energy minima nor TS structures, only the ΔE_int_ + ΔG(solv) term could be reasonably compared.

The theoretical results could be summarized as follows. The X=C-Y-CH_3_ *cis* conformers are more stable than the *trans* forms for all four studied esters in the gas phase. ΔE_int_ is considerably larger for OCH_3_ esters compared with SCH_3_ esters. The B97D/aug-cc-pvtz level, however, probably underestimates the *cis-trans* energy difference for methyl acetate as of ΔH = ΔE_int_ + ΔH_th_ = 5.71 − 0.07 = 5.64 kcal/mol. Here ΔH_th_ is the thermal enthalpy correction (including relative zero-point energies) at T = 298 K° and p = 1 atm. The total relative free energy is remarkably larger, 5.71 + 0.89 = 6.60 kcal/mol. Blom and Günthard [36] estimated the enthalpy difference as 8.5 ± 1 kcal/mol and commented that there was a very small band in the matrix IR spectra, and “it is possible that this band is due to the methyl acetate”. The equilibrium composition depends on the relative total free energy. If, as calculated here, ΔG_tot_ is as large as 6.6 kcal/mol, the trans conformer could be hardly observed. Pyckhout *et al*. [34] calculated ΔE = 10.3 kcal/mol at the *ab initio* 4-21G level. Nagy *et al*. [7] obtained ΔH = 7.8–8.2 kcal/mol at the MP2 and B3LYP levels, using the 6-311++G** basis sets, whereas the G3B3 [37] ΔH value by Terhorst and Jorgensen is 7.46 kcal/mol [8]. Although the aug-cc-pvtz basis set was applied presently in geometry optimizations, which is considerably larger than in any former study, the author has no good explanation for the underestimation of ΔH for methyl acetate.

**Table 3:** Relative energies for esters as the function of the rotation about the C-O and C-S bonds ^a^.

	Gas	Chloroform	Acetonitrile
	ΔE_int_	ΔE_int_ + ΔG(solv)	ΔE_int_ + ΔG(solv)
O=C-O-C			
0	0.0	0.0 + 0.0 = 0.0	0.0 + 0.0 = 0.0
30	2.88	2.91 − 0.04 = 2.87	2.95 − 0.12 = 2.83
60	8.76	8.85 − 0.13 = 8.72	8.96 − 0.35 = 8.61
TS(95.4, 92.2, 90.7)	12.40	12.59 − 0.75 = 11.84	12.80 − 1.29 = 11.51
120	11.15	11.54 − 1.57 = 9.97	11.98 − 2.61 = 9.37
150	7.00	7.96 − 2.48 = 5.48	8.59 − 4.02 = 4.57
180	5.71	6.37 − 2.86 = 3.51	7.09 − 4.59 = 2.50
ΔG_th_	0.89	1.09	1.10
ΔG_th_(TS)	0.44	0.48	0.40
O=C-S-C			
0	0.0	0.0 + 0.0 = 0.0	0.0 + 0.0 = 0.0
30	2.72	2.76 + 0.02 = 2.78	2.80 − 0.03 = 2.77
60	8.86	8.93 − 0.12 = 8.81	9.00 − 0.32 = 8.68
90	12.16	12.34 − 0.61 = 11.73	12.55 − 1.17 = 11.38
120	10.27	10.60 − 1.10 = 9.50	10.89 − 1.87 = 9.02
150	5.63	6.08 − 1.62 = 4.46	6.57 − 2.72 = 3.85
180	3.35	3.86 − 1.89 = 1.97	4.42 − 3.12 = 1.30
ΔG_th_	1.18	1.29	1.31
S=C-O-C			
0	0.0	0.0 + 0.0 = 0.0	0.0 + 0.0 = 0.0
30	2.83	2.84 + 0.00 = 2.84	2.86 − 0.04 = 2.82
60	8.95	8.98 + 0.00 = 8.98	9.02 − 0.11 = 8.91
90	12.94	13.04 − 0.35 = 12.69	13.17 − 0.72 = 12.45
120	11.61	11.94 − 1.37 = 10.57	12.33 − 2.32 = 10.01
150	7.81	8.44 − 2.64 = 5.80	9.14 − 4.30 = 4.84
180	6.25	7.00 − 3.19 = 3.81	7.83 − 5.13 = 2.70
ΔG_th_	0.10	0.42	0.51
S=C-S-C			
0	0.0	0.0 + 0.0 = 0.0	0.0 + 0.0 = 0.0
30	3.02	3.00 − 0.15 = 2.85	3.03 − 0.28 = 2.75
60	9.96	9.86 + 0.12 = 9.98	9.76 + 0.16 = 9.92
TS(90.6, 89.7, 89.0)	14.30	14.27 + 0.07 = 14.34	14.24 − 0.10 = 14.14
120	11.40	11.52 − 0.93 = 10.59	11.71 − 1.55 = 10.16
150	5.60	6.03 − 1.92 = 4.11	6.62 − 3.20 = 3.42
180	3.10	3.65 − 2.05 = 1.60	4.30 − 3.42 = 0.88
ΔG_th_	0.16	0.80	1.10
ΔG_th_(TS)	0.47	0.85	1.16

^a^ Energies in kcal/mol, torsion angles in deg. Local-energy-minima torsion angles are underscored.

**Table 4:** Torsion barrier energies relative to the *cis* conformation in the gas phase ^a^.

	CH_3_COOCH_3_	CH_3_COSCH_3_
B3LYP/6-311++G** ^b^	13.35	11.80
B97D/aug-cc-pvtz ^c^	12.40	12.16
MP2/6-311++G** ^b^	13.45	11.18
MP2/aug-cc-pv(t+d)z		11.98
INDO ^d^	29.10	14.06
PM3		6.05
PM6		6.53
PDDG/PM3		4.87

^a^ Energies in kcal/mol. ^b^ Ref. [7]. ^c^ Plus a set of “d” functions for the S-methyl thioacetate. ^d^ Rigid rotation, spd basis for S-methyl thioacetate, ref. [38].

Della Vedova found only one conformation in the sample of CH_3_COSCH_3_, assigning it to the *cis* conformation [35]. Table 3 predicts ΔG_tot_ = 4.53 kcal/mol for the *trans* conformer (4.60 kcal/mol in ref. [8]), a value large enough for preventing the presence of this conformer at an experimentally observable concentration in the gas-phase mixture. Overall, gas-phase calculation results suggest that ΔG_tot_ is prohibitively large for the observation of the *trans* CH_3_ position in any studied X=C-Y- CH_3_ ester moiety. The question is, whether the solvent effects could modify this equilibrium ratio.

The conclusion about the solvent effect for esters (always talking about the four studied prototypes) is that the *cis* conformer remains the prevailing conformation, although the *cis-trans* free energy difference could be largely decreased in comparison with the gas phase. The 0° and 180° torsion angles are underscored in Table 3, indicating local energy minima both in chloroform and the acetonitrile solvents. Adding ΔG_th_ to the 180° ΔE_int_ + ΔG(solv) value, the obtained ΔG_tot_ (in the order of the appearance of the structures in Table 3) are 4.60, 3.26, 4.23, and 2.40 kcal/mol in chloroform, and 3.60, 2.61, 3.21, and 1.98 kcal/mol in acetonitrile. The in-acetonitrile values are consistently smaller than those with chloroform solvent, but even the smallest calculated ΔG_tot_ value for CH_3_CSSCH_3_ in acetonitrile allows for a *trans-cis* ratio of only about 4:96 at T = 298 K. This ratio was calculated by using the relationship −RT ln K = ΔG°, where ΔG° was accepted to be equal to the calculated ΔG_tot_, and the concentration related activity coefficients were accepted to be equal for the two conformers in very dilute solution as modeled by IEF-PCM. [39,40]. Thus one may conclude that the effect of a non-protic solvent on the *cis-trans* equilibrium of esters is small, but may not be negligible for esters mainly with the –SCH_3_ group.

Another important question is: how can the esters reach the equilibrium composition? For compounds with a polar hydrogen atom, even conformational equilibration could be reached by the catalytic involvement of a protic solvent [41,42]. For esters in a non-protic solvent, this reaction path does not seem to be travelable. The straightforward mechanism is, of course, the rotation about the C-Y (Y = O, S) bond. This is a simple mechanism if the barrier to the rotation is energetically affordable. The transition states were identified for CH_3_COOCH_3_ and CH_3_CSSCH_3_ in all three environments, and the energy values for the barrier scatter in the 11.5–14.3 kcal/mol range. The barrier tops are close to 90°. The activation free energy for passing the barrier can be obtained by adding ΔG_th_(TS) to the TS energy, and subtracting 0.41 kcal/mol for the entropy of mixing for the TS optical antipodes. Still one is left in the range of ΔG_tot_ = 11–15 kcal/mol. For the other two molecules, the 90° relative energies are also in this latter range. This activation free energy may be considered fairly large, although, e.g., Lunazzi *et al*. [43] found experimentally the successful annular tautomerizations of azoles in DMSO, THF and CD_2_Cl_2_, with activation free energies of 11–14 kcal/mol. Even though the chemical problem studied by Lunazzi *et al*. is quite different, an activation free energy in the indicated range seems to be affordable for structural transformations in solution. Then this author considers the *cis-trans* equilibration for simple esters in solution via the rotation of the methyl group about the C-Y bond.

Components of the ΔE_int_ + ΔG (solv) term as the function of the X=C-Y-CH_3_ torsion angle are provided in the 0–180° range in Table 3. ΔE_int_, compared with the corresponding gas-phase value, changes only slightly up to about 90° torsion, but conspicuously and unanimously increases after then in solution in comparison with the gas phase. What could be the explanation?

The PCM optimization procedure (keeping only the X=C-Y-CH_3_ torsion angle at the set value with the exception for the local-minimum-energy conformers) seeks the structure corresponding to the minimum of the E_int_+G_elst_ term. The solvent polarizes the solute’s electron distribution and slightly modifies the geometry throughout the optimization. The internal energy itself, E_int_ necessarily increases, whereas a more and more negative G_elst_ is being developed in parallel, and the E_int_+G_elst_ minimum for the considered conformer is ultimately reached. As long as ΔG(solv) is only of a few tenths of a kcal/mol for different conformers as calculated in the 0–60° torsion range, ΔE_int_ changes only moderately compared with it in the gas phase. However, the stiffness of the PES depends on the course of ΔE_int_ in the given environment and could be considered moderate only up to about 30°. ΔE_int_ is here about 3 kcal/mol for all studied esters both in the gas phase and solution, but increases to 8.8–10.0 kcal/mol at torsion angle of 60°.

ΔE_int_(solution), as the relative internal energy in solution, could both increase and decrease compared with ΔE_int_(gas) depending on the sensitivity of the internal energy itself on the solvent effect for the *cis* conformer compared with a torsioned structure. When, however, ΔG_elst_ can become remarkable, due to, e.g., the increase of dipole moment, the molecular structure gets distorted accordingly. Table 3 shows that ΔG(solv) is at least about –1 kcal/mol starting at X=C-Y-CH_3_ = 120°.

Overall, the question can be raised: how reliable is the calculated torsion potential curve for ΔE_int_? This is an important issue, since molecular modelers may want to develop torsional potential parameters for esters/ethers to be utilized in Monte Carlo and/or molecular mechanics/dynamic theoretical binding studies. Unfortunately, no experimental torsion barrier data are available for the molecules under scrutiny. To get some information about the stability of the calculated data, MP2/aug-cc-pv(t+d)z calculations in addition to the B97D calculations have been carried out for the *cis* and 90° rotated CH_3_COSCH_3_ structures in the gas phase and are compared with PM3, PM6 and PDDG/PM3 results based on symmetry-unrestricted geometry optimizations. Former INDO [38] and B3LYP/MP2 studies [7] have been also considered.

INDO geometries are fairly good for the *cis* CH_3_COOCH_3_, but cannot be reasonably judged for CH_3_COSCH_3_, where the authors did not optimize the S-C bond length and the C-S-C bond angle. Using the sp basis set, the C(O)-S bond length was underestimated by 0.05 Å, with the spd set the C=O became even further away from the experimental value. In the torsion energy barrier calculation they considered a rigid rotation, whereas Table 2 of this paper shows that the C-S bond length increases by about 0.09 Å. PM3 finds the C-S bond length longer than that for S-C and the CSC angle is too large by about 6° for the *cis* ester conformer. With PM6, the two C-S bonds are still only equally long, the CSC angle is too large by 4°, and the CCO angle was overestimated by about 5°. All these parameters are close to the experimental values from PDDG/PM3 optimizations, although the *trans* hydrogens of the ester methyl group was found 23° out of the heavy-atom plane. Furthermore, all three optimizations predicted eclipsed HCCO arrangements in contrast to the experimental. 

Still the main problem is related to the predicted torsion barrier. The results indicate (Table 4) that the DFT and MP2 energy barriers are similar; they scatter in a range of about 1 kcal/mol. In contrast, the INDO barrier is about the double of the DFT/MP2 values for CH_3_COOCH_3_, whereas PM3, PM6, PDDG/PM3 give barrier heights about the half of the DFT/MP2 values. All these findings together suggest that consistent, numerically stable potential curves could be probably only obtained based on large-basis-set calculations.

### 3.3. Energy Results for Ethers

Relative energies are provided in Table 5 with respect to the actually lowest-energy conformer. In contrast to esters, the relative energy/free energy of two local energy minimum structures are relatively small, up to about 2 kcal/mol. The solvent can both increase (OCH_3_) and decrease (SCH_3_) the energy separations for the local-minimum-energy ether structures in comparison with the gas phase. The X-C-Y-C torsional angle in the lowest-energy conformation is 180° for the 2-OCH_3_ ethers. The second local energy minimum is, however, largely different: XCOC is about 50° and 6° for the furan and thiophene derivative, respectively. The most stable conformation for the furan-2SCH_3_ thioether is a *gauche* form with OCSC = 69–76°, whereas for the second stable structure OCSC = 180°. The most complicated equilibrium was found for the thiophene-2SCH_3_ thioether. None of the stable conformers adopt a coplanar heavy-atom arrangement. As mentioned above, if conformers with a molecular symmetry plane, as in this case with XCYC torsion angles of 0° and 180°, do not correspond to local energy minima, then they must exhibit a transition state structure due to the equivalence of the clockwise and anti-clockwise rotation. For the 2-SCH_3_ thiophene, in-solution minimum-energy structures adopt XCYC torsion angles of 83–84° and ~172°. There must be a local energy maximum around 120°–150° (see Table 5), thus this potential curve has two minima and three TS structures in the 0–180° range of the XCYC rotation. The subtle effect of the solvents indicates that the marginal ΔE_int_+ΔG(solv) preference for the two minimum-energy structures is reversed in the two solvents.

In general, ΔG_tot_ is small for any studied ether in solution. Considering ΔG_th_ and –0.41 kcal/mol for the entropy of mixing for non-planar structures, ΔG_tot_ for the less stable conformer is (in the order of the structure appearance in Table 5) 0.34, 1.11, 0.39, and 0.64 kcal/mol in chloroform, and 1.10, 1.40, 0.24, and 0.58 kcal/mol in acetonitrile. Considering the moderate rotational barriers, equilibration of the stable conformers via C-Y rotation at T = 298 K° would not be hindered. Provided the thermodynamic control for the conformer equilibration, structures with different orientations for the lone pairs of the O and S atoms in the methyl ether and thiomethyl ether groups are expected in the solution. This is a remarkable difference in comparison with esters, where the prevailing lone pair arrangements are determined by the XCYC cis conformation. For esters, the lone pairs of the X atom point approximately toward northwest and east, and those for Y about toward southwest if the CH_3_-C axis points from west to east (Figure 1). Thus the lone pairs point in fairly opposite directions in this case. For furan and thiophene ethers, the lone pair of the X heteroatom (nearly) in the ring plane points south. Due to fairly unhindered rotation of the CH_3_ group about the C-Y bond, the lone pairs of Y would point in many different directions, including orientations basically parallel or antiparallel with those on X. This variety of the lone pair directionality could be expediently utilized through drug design, as hinted for in the Introduction section.

**Table 5:** Relative energies for ethers as the function of the rotation about the C-O and C-S bonds ^a^.

	Gas	Chloroform	Acetonitrile
	ΔE_int_	ΔE_int_ + ΔG(solv)	ΔE_int_ + ΔG(solv)
Furan, O-C-O-C			
0	1.53	1.38 + 0.72 = 2.10	1.18 + 1.16 = 2.32
45.1	1.43		
49.9		1.31 + 0.74 = 2.05	
53.9			1.18 + 1.11 = 2.29
90	2.12	2.05 + 0.58 = 2.63	1.97 + 0.79 = 2.76
120	2.41	2.39 + 0.23 = 2.62	2.37 + 0.27 = 2.64
150	1.11	1.11 + 0.05 = 1.16	1.11 + 0.04 = 1.15
180	0.0	0.0 + 0.0 = 0.0	0.0 + 0.0 = 0.0
ΔG_th_		−1.30	−0.78
Furan, O-C-S-C			
0	1.92	1.83 − 0.06 = 1.77	1.70 + 0.10 = 1.80
30	1.14	1.06 +0.03 = 1.03	0.95 + 0.19 = 1.14
69.2	0.0		
73.7		0.0 + 0.0 = 0.0	
75.9			0.0 + 0.0 = 0.0
90	0.31	0.32 − 0.09 = 0.23	0.33 − 0.15 = 0.18
120	1.35	1.39 − 0.35 = 1.04	1.45 − 0.54 = 0.91
150	1.45	1.49 − 0.60 = 0.89	1.56 − 0.85 = 0.71
180	0.92	0.96 − 0.60 = 0.36	1.01 − 0.80 = 0.21
ΔG_th_		0.34	0.78
Thiophene, S-C-O-C			
0	0.87	0.81 + 0.17 = 0.98	0.70 + 0.36 = 1.06
6.3		0.81 + 0.15 = 0.96	
5.1			0.70 + 0.35 = 1.05
30	1.10	1.04 + 0.22 = 1.26	0.94 + 0.40 = 1.34
60	1.58	1.52 + 0.03 = 1.55	1.44 + 0.60 = 2.04
90	1.89	1.86 + 0.05 = 1.91	1.80 + 0.49 = 2.29
120	1.98	1.97 − 0.03 = 1.94	1.95 + 0.29 = 2.24
150	0.90	0.90 − 0.01 = 0.89	0.89 + 0.0 = 0.89
180	0.0	0.0 + 0.0 = 0.0	0.0 + 0.0 = 0.0
ΔG_th_		−0.16	−0.40
Thiophene, S-C-S-C			
0	1.10	1.06 − 0.52 = 0.54	1.01 − 0.55 = 0.46
30	0.78	0.75 − 0.32 = 0.43	0.70 − 0.34 = 0.36
60	0.21	0.19 − 0.10 = 0.09	0.16 − 0.08 = 0.08
82.7	0.0		
83.3		0.0 + 0.0 = 0.0	
83.8			0.0 + 0.0 = 0.0
120	0.66	0.69 − 0.29 = 0.40	0.72 − 0.42 = 0.30
150	0.99	1.02 − 0.59 = 0.43	1.08 − 0.82 = 0.26
171.9		0.85 − 0.73 = 0.12	
172.1			0.88 − 0.97 = -0.09
180	0.81	0.84 − 0.67 = 0.17	0.88 − 0.92 = -0.04
ΔG_th_		0.52	0.67

^a^ Energies in kcal/mol, torsion angles in deg. See also the footnotes for Table 3.

### 3.4. Atomic Charges

In molecular mechanics, Monte Carlo or molecular dynamics studies utilizing some effective pair potential for calculating the interaction energy for a ligand-macromolecule complex, one of the most important issue is the proper estimation of the electrostatic component. This energy is generally represented, at least as the leading term of an expanded series, by the sum of Coulomb energies of effective net atomic charges. There are a number of different methods known in the literature for deriving these charges, and the applied force field parameters must be harmonized with the actually derived charge sets. The charges presented in Table 6 were derived by means of the CHELPG procedure [44], fitting the in-solution atomic charges to the corresponding molecular electrostatic potential.

**Table 6:** Net atomic charges fitted to the B97D/aug-cc-pv(t+d)z molecular electrostatic potential by means of the CHELPG procedure ^a^.

	Gas						CHCl_3_			CH_3_CN		
	X	Y				DM	X	Y	DM	X	Y	DM
CH_3_C(O)OCH_3_												
*cis*	−0.55		−0.40			1.87	−0.61	−0.41	2.33	−0.63	−0.42	2.53
*trans*	−0.55		−0.39			4.43	−0.63	−0.43	5.52	−0.66	−0.44	5.99
CH_3_C(O)SCH_3_												
*cis*	−0.45	−0.25				1.30	−0.51	−0.26	1.71	−0.53	−0.26	1.93
*trans*	−0.45	−0.19				4.16	−0.52	−0.23	5.25	−0.55	−0.25	5.74
CH_3_C(S)OCH_3_												
*cis*	−0.32	−0.29				2.32	−0.39	−0.30	3.07	−0.42	−0.30	3.41
*trans*	−0.33	−0.35				4.67	−0.43	−0.38	6.15	−0.47	−0.39	6.80
CH_3_C(S)SCH_3_												
*cis*	−0.28			−0.16		2.01	−0.35	−0.15	2.84	−0.39	−0.13	3.27
*trans*	−0.26			−0.15		4.35	−0.35	−0.17	5.85	−0.40	−0.17	6.55
Furan-2OCH_3_												
OCOC = 45–54°	−0.14			−0.37		1.13	−0.15	−0.41	1.35	−0.16	−0.43	1.49
OCOC = 180°	−0.19			−0.36		1.94	−0.21	−0.39	2.40	−0.23	−0.40	2.62
Furan-2SCH_3_												
OCSC = 69–76°	−0.17			−0.25		1.56	−0.20	−0.30	2.04	−0.21	−0.33	2.29
OCSC = 180°	−0.13			−0.17		1.95	−0.15	−0.21	2.50	−0.17	−0.23	2.77
Thiophene-2OCH_3_												
SCOC = 0–6°	−0.04			−0.31		1.11	−0.05	−0.34	1.30	−0.04	−0.35	1.37
SCOC = 180°	−0.03			−0.26		1.85	−0.05	−0.28	2.29	−0.06	−0.29	2.51
Thiophene-2SCH_3_												
SCSC = 83–84°	−0.03	−0.24			1.63		−0.03	−0.30	2.10	−0.03	−0.32	2.33
SCSC = 171–172°	0.01	−0.14			1.86		−0.02	−0.19	2.36	−0.02	−0.20	2.61

^a^ Charges in atomic charge units, DM is the dipole moment in debye.

^a^ Charges in atomic charge units, DM is the dipole moment in debye.

The derived charges are, however, fitting method and theoretical level dependent. In a recent publication by Nagy *et al*. [27], the CHELPG charges were compared for 3,5-dimethoxy-1,2,4-thiadiazole calculated in tetrahydrofuran solvent. Non-negligible differences were noticed in the charges derived at different theoretical levels, although the trends were generally preserved. Monti and Nagy [21,45] compared CHELPG and RESP [46] atomic charges for the trimethyl- and dimethylammonium cations at different theoretical levels. Non-negligible differences were noticed in some cases also in these studies. These findings confirm that the charge derivation by different methods and/or at different theoretical levels generally lead more or less different charge sets. Charges in Table 6 were derived on the basis of large-basis-set calculations, where the molecular electrostatic potential utilized in the atom-charge fitting process was generated by means of a wave function reflecting the electron correlation for the target molecule. Although the present study represents high-level calculations and probably provides fairly relevant charges, the primary goal of the comparison in Table 6 is to point out the *sensitivity* of the obtained charges to the conformation and solvent effects.

A physically measurable structure characteristic is the dipole moment (DM). No proper experimental data have been found for the present molecules, the only available experimental value is 1.72 ± 0.09 D for the liquid methyl acetate, probably assignable to the *cis* form [47]. Table 6 shows that the quantum-mechanical DM is both conformation and environment dependent. The dipole moment gradually increases when the dielectric constant of the solvent increases compared to the gas phase (with unit dielectric constant). This finding emphasizes the polarization effect of the environment because geometries have only slightly changed upon solvation. The conformation is inherently a major determinant of the molecular dipole, as reveals from their large changes mainly for esters.

The dipole moment is capable only for an overall characterization of the molecule in the given environment, whereas effective pair-potentials need individual atom charges. It is to be emphasized, however, that atom charges have no physical meaning; they facilitate a simple way to calculate molecular electrostatic interactions in the system. It is important to know, how well they would reproduce the overall, physically meaningful dipole moment of the molecule calculated quantum mechanically. Not indicated in Table 6, but the dipoles calculated by means of net atomic charges deviate by less than 0.13 D from the corresponding exact B97D/aug-cc-pvtz values (as an exception, the difference amounted to 0.17 D for 2SCH_3_ thiophene in solution). Thus the dipoles calculated by means of net atomic charges approach the exact values satisfactorily in general, crediting the underlying atom-charge distribution.

Changes in the corresponding atomic charge values (if the change is larger than 0.01 units thus larger than the rounding error) are gradual: from left to right the negative charges monotonically vary; generally increase in accord with the increasing dipole moments. The trend in the change for a specific atom in different conformations (upper line compared to the lower line) is generally the same in all three environments. All these findings together indicate that one has to consider conformation dependent charges, and they have to be determined in the specific solvent for providing Monte Carlo or molecular dynamics atom-charge parameters for explicit solvent simulations.

### 3.5. Vibrational Frequencies

Hernandez *et al*. [48] presented some parts of the IR spectra for methyl acetate and its deuterated forms recorded in dilute CCl_4_. These authors also performed 6-31** theoretical calculations, and assigned the six C-H stretching frequencies and IR intensities to the acetyl methyl and the ester methyl groups. The dielectric constant of the used solvent is 2.23 [47], thus the results could be relevantly compared with our calculated C-H stretching frequencies for the gas phase and in chloroform solution with ε = 4.71.

The presently calculated B97D frequencies for CH_3_COOCH_3_ and CH_3_CSSCH_3_, both for their *cis* and *trans* conformations are summarized in Table 7. The X=C-Y-C torsion frequencies for the *cis* forms are also provided. The corresponding vibrations in the *trans* forms are strongly coupled with both methyl torsions.

**Table 7:** B97D frequencies and IR intensities for CH_3_COOCH_3_ and CH_3_CSSCH_3_^a^.

	Gas		CHCl_3_		CH_3_CN		Exp ^b^
	ω	Int	ω	Int	ω	Int	
CH_3_COOCH_3_, *cis*							
C-H stretching							
acetyl methyl	3088	8.3	3080	9.2	3077	5.2	2844–3026
	3041	6.5	3034	3.2	3031	1.9	
	2967	5.8	2960(c)	5.0	2956(c)	13.3	
ester methyl	3080	15.3	3079	10.2	3079	12.3	
	3045	23.1	3046	20.8	3046	20.1	
	2961	33.0	2957	26.5	2955(c)	15.5	
torsional							
O=C-O-CH_3_	177	6.0	196	8.4	197	9.5	
CH_3_COOCH_3_, *trans*							
C-H stretching							
acetyl methyl	3092	6.0	3085	4.3	3082	3.4	
	3031(c)	14.7	3026	0.9	3022	0.3	
	2961	7.7	2955	3.0	2952(c)	4.4	
ester methyl	3067	18.5	3073	13.5	3074	11.6	
	3020(c)	24.8	3036	22.5	3041	15.9	
	2941	35.6	2948	27.9	2949	21.0	
CH_3_CSSCH_3_, *cis*							
C-H stretching							
acetyl methyl	3042	5.1	3032	7.4	3027	10.7	
	3025	9.4	3022	8.5	3021	5.3	
	2945	9.4	2939	3.4	2936	1.9	
ester methyl	3070	4.0	3064	1.9	3061	1.0	
	3063	3.0	3058	1.4	3056	0.8	
	2969	11.7	2953	5.0	2950	2.8	
torsional							
S=C-S-CH_3_	220	4.3	191	7.3	188	8.3	
CH_3_CSSCH_3_, *trans*							
C-H stretching							
acetyl methyl	3069	3.7	3063(c)	2.8	3061(c)	2.8	
	3003	7.8	2999	2.7	2995	0.8	
	2938	10.5	2933	2.6	2929	0.3	
ester methyl	3064	5.6	3062(c)	2.7	3061	0.6	
	3061	6.9	3062(c)	2.5	3060(c)	1.8	
	2960	2960	2955	5.2	2952	1.9	

^a^ Frequencies (ω) in cm^−1^. Letter “c” in parentheses refers to non-negligible coupling with displacements in the other methyl group. ^b^ Ref. [48], data recorded in dilute CCl_4_ solution.

Our frequencies have been calculated in the harmonic oscillator approximation [22]. As it is known, the calculated high-frequency stretching values are generally larger than the anharmonic frequencies [28], which are probably observed in the experimental spectra. Then it is not surprising that the present C-H stretching frequencies are higher by 60–120 cm^−1^ than the experimental values. Although Cappelli *et al*. [49] developed recently a perturbative methodology considering nonequibrium solvation conditions for evaluating anharmonic vibrational frequencies in solution, the method is not available in Gaussian 09. Nonetheless, assignment of the C-H stretching frequencies to vibrations of the two methyl groups is still possible.

Solvation generally results in a gradual decrease of the C-H frequencies. Shifts in ω are up to about 20 cm^−1^. However, these frequencies for the ester methyl group of CH_3_COOCH_3_ are hardly sensitive to the solvent effects in the *cis* conformation, and remarkably increase in the *trans* form with increasing solvent polarity. No such “out of trend” behavior has been calculated for CH_3_CSSCH_3_, where, however, the ester group shows insensitivity for the solvent effects in the *trans* conformation.

In accord with the conclusions of Hernandez *et al*. above, the IR intensities of the C-H stretching frequencies are consistently higher for the ester rather than for the acetyl methyl group for CH_3_COOCH_3_. This conclusion is valid for the series calculated both for the *cis* and the *trans* conformers, both in the gas-phase and in solution. For CH_3_CSSCH_3_, no such clear-cut trend was calculated, the IR intensities are of the same order of magnitude for the C-H vibrations of the two methyl groups. In comparison with the experiment for CH_3_COOCH_3_ it has to be mentioned, however, that the largest intensities were observed in the middle of the spectrum, whereas the largest intensities were theoretically calculated at the low-frequency site both here and in ref. [48].

The X=C-Y-CH_3_ torsional frequencies were assigned to 177–197 cm^−1^ vibrations for CH_3_COOCH_3_ and to 188–220 cm^−1^ for CH_3_CSSCH_3_. The IR intensities increase for both molecules in their *cis* form with increasing polarity of the environment. However, the theoretical frequencies increase in this series for CH_3_COOCH_3_ but decreases for CH_3_CSSCH_3_, further emphasizing the structural differences for XY=OO and XY=SS esters.

## 4. Conclusions

Simple aliphatic esters and 2-substituted heterocyclic ethers have two local-energy-minimum conformations when the methyl group of the XCYCH_3_ moiety (X, Y = O, S) is rotated from 0 to 180°. Due to molecular symmetries, clockwise and anticlockwise rotations in the indicated torsional energy range are equivalent. The minimum-energy torsion angles could deviate by up to about 10° when the gas-phase molecule is dissolved in chloroform or acetonitrile. Bond lengths and bond angles change by up to 0.017 Å and a few degrees, respectively, as calculated at the B97D/aug-cc-pv(t+d)z theoretical level (d orbitals only for S containing molecules) and considering the IEF-PCM continuum dielectric solvent approximation for the in-solution geometry optimization.

In contrast to the small effect on the geometry, the solvent has remarkable effects on relative conformer energies for the stable species. Although the prevailing conformation is always *cis* for the studied esters with practically coplanar heavy atom skeleton, the energy separations of the *cis* and *trans* conformers decrease by 1–3 kcal/mol in solution compared with the gas phase. The smallest free energy difference was calculated at 1.98 kcal/mol, allowing for a *trans-cis* ratio of 4:96 at T = 298 K°. The barriers for the rotation about the C-Y bonds are in the range of 11–15 kcal/mol, which can be overridden as concluded from comparisons with experimental results found for successful in-solution equilibration for azoles. Thus one may expect that esters could conformationally equilibrate under the thermodynamic control.

2-Methoxy and 2-thiomethyl furan and thiophene ethers exhibit also two local-energy-minimum conformations for which the methyl group can have different favorable rotational positions in the 0–180° range for the XCYCH_3_ torsion angle. The aromatic rings take practically coplanar structures, whereas the methyl group favors XCYCH_3_ = 0–6° *cis*, 45–84° *gauche*, and 172–180° *trans* conformations in different environments for different ethers. The two locally stable conformers are separated in free energy only by 0.2–1.4 kcal/mol, and the barrier to rotation is about 2–3 kcal/mol at most. Thus conformer equilibration is also feasible under thermodynamic control. The various favorable ether conformations allow different relative orientations of the lone pairs for the ring heteroatoms and the 2Y (Y = O,S) atoms from nearly parallel to antiparallel combinations. In contrast, the lone pair orientations are fairly antiparallel in the almost exclusively prevailing cis conformation for esters. This possible difference of the lone-pair orientations for ethers and esters could be deliberately utilized in structure based drug design in order to provide favorable hydrogen-bond acceptor sites in a protein binding cavity. Furthermore, the distance of the head atoms in a ligand-protein hydrogen bond could be remarkably changed by the replacement of an O atom by an S atom.

Solvent effects are considerable on the dipole character of both esters and ethers. With increasing polarity for the solvent, the dipole moment monotonically increases in comparison with the gas-phase value. Molecular electrostatic potential fitted atomic charges reproduce well the quantum mechanical, exact dipole values. Since the derived charges show conformation and solvent dependence, charges for each conformer should be separately fitted in the specific solvent in order to obtain reliable charge parameters for Monte Carlo or molecular dynamics, explicit solvent simulations.

In summary, this study has called attention to the need for theoretical exploration of possible structural differences for chemical systems with formal substructure similarity. The conformation analysis has revealed large differences for the pairs of the corresponding esters and ethers with equal XCYC substructures. The obtained results could usefully be applied throughout the structure-based design of a related drug candidate.

## Supplementary Materials

Detailed optimized geometric parameters for the CH_3_CSOCH_3_ and CH_3_CSSCH_3_ molecules in the gas-phase, chloroform, and acetonitrile are provided.

Supplementary materials can be accessed at: http://www.mdpi.com/1420-3049/18/7/8063/s1.
