# Supplementary material for: In-Solution Conformational Analysis of the XCYCH3 Moiety for Small Esters and Ethers with all Combinations of X, Y = O, S
Source: Molecules. 2013 Jul 8;18(7):8063–82. doi: 10.3390/molecules18078063 (PMC6270607; doi:10.3390/molecules18078063)
Supplement: Supplementary file 1 [file molecules-18-08063-s001.pdf]

Supplementary Materials

Table S1. Geometric parameters optimized at the B97D/aug-cc-pv(T+d)z levels for *O*-methyl ethanethioate (O-methyl thioacetic acid) and dithioacetic acid methyl ester [a].

	**Gas**	**$CHCl_3$**	**CH_3CN**
**CH_3CSOCH_3, *cis***			
C-C	1.509	1.507	1.506
C=S	1.643	1.650	1.654
C-O	1.345(1.375) [b]	1.338(1.368)	1.335(1.367)
$O-CH_3$	1.447	1.451	1.453
C-C=S	125.1	124.9	124.8
S=C-O	125.7	125.5	125.5
$C-O-CH_3$	119.1(115.4)	119.7(115.5)	120.0(115.6)
**CH_3CSOCH_3, *trans***			
C-C	1.510	1.506	1.503
C=S	1.638	1.650	1.655
C-O	1.353	1.342	1.338
$O-CH_3$	1.446	1.455	1.459
C-C=S	124.2	124.1	124.1
S=C-O	118.6	118.2	118.0
$C-O-CH_3$	120.7	120.6	120.6
**CH_3CSSCH_3, *cis***			
C-C	1.518	1.517	1.516
C=S	1.643	1.650	1.653
C-S	1.747(1.830)	1.739(1.829)	1.735(1.828)
$S-CH_3$	1.811	1.811	1.811
C-C=S	122.6	122.4	122.3
S=C-S	126.0	126.0	126.0
$C-S-CH_3$	104.0(97.5)	104.8(97.9)	105.1(97.9)
**CH_3CSSCH_3, *trans***			
C-C	1.510	1.507	1.506
C=S	1.643	1.653	1.658
C-S	1.747	1.739	1.734
$S-CH_3$	1.832	1.829	1.828
C-C=S	124.0	123.8	123.7
S=C-S	117.9	117.9	117.8
$C-S-CH_3$	106.2	106.2	105.3

[a] Distances in Å, angles in degrees. Cis and trans structures correspond to S=C-X-C (X=O, S) torsional angles of ~0° and ~180°, respectively. For CH_3CSOCH_3, the symmetry is nearly C_s in both conformations and a methyl hydrogen nearly eclipses the tiocarbonyl sulfur. The HCC=S torsional angle for the nearly in-plane H of the cis CH_3CSSCH_3 ester is 172-175-177° in gas, $CHCl_3$ and CH_3CN, respectively. The symmetry is practically C_s for the trans conformer, a methyl hydrogen eclipses nearly the thiocarbonyl sulfur.
[b] Values in parentheses stand for parameters with largest changes in the transition state geometry of for the XCYC = 90° structures.
